# Eruptive lentiginosis confined to areas of regressing psoriatic plaques after adalimumab treatment^☆^^☆☆^

**DOI:** 10.1016/j.abd.2020.05.011

**Published:** 2020-11-18

**Authors:** Fernando Garcia-Souto

**Affiliations:** Deparment of Dermatology, Valme University Hospital, Seville, Spain

*Dear Editor,*

Eruptive lentiginosis confined to areas of regressing psoriatic plaques is a rare phenomenon. Initially described after phototherapy, several other treatment regimens used in psoriasis including topical and systemic biologic agents, have been reported to induce lentigines.^1^ The author reports a new case of eruptive lentiginosis following treatment with adalimumab.

A 45-year-old female with no relevant medical history presented to this department with multiple brownish lesions in areas previously occupied by psoriatic plaques seven months after initiating adalimumab. She denied having applied any topical treatment or having received any sun exposure. Physical examination revealed grouped brown macules over previously affected areas (Fig. 1). The patient had suffered from chronic plaque psoriasis since adolescence, proven to be refractory to topical therapies, methotrexate, and cyclosporine. Phototherapy was not performed in this patient. She was not taking any other medications. A punch skin biopsy showed hyperpigmentation of the basal layer, consistent with lentigo. No treatment was initiated due to patient refusal. The lesions remained stable throughout one year of follow-up.

**Figure 1:**
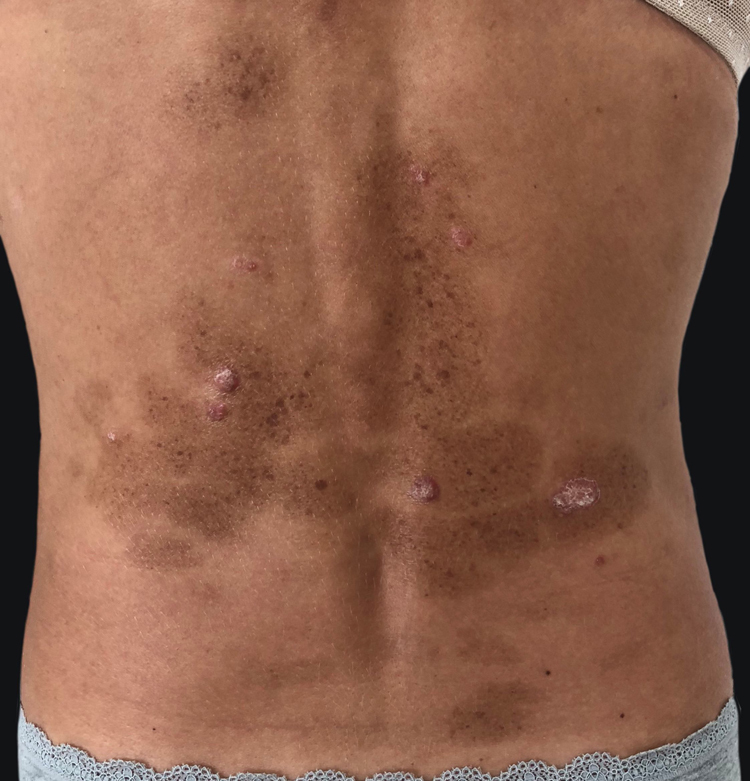
Multiple brownish macules in areas previously occupied by psoriatic plaques.

Lentigos confined to resolved psoriatic plaques have been rarely mentioned in the literature. The literature features reports following topical treatments and biological therapies used in psoriasis. Among biological therapies, eruptive lentiginosis has been reported in relation to infliximab, adalimumab, etanercept, ustekinumab and secukinumab. To the best of the author’s knowledge, to date there is only one case reported associated with classic systemic therapies.^2^

The pathophysiology is not well documented. Some cytokines produced in psoriatic skin are known to stimulate melanogenesis and might be responsible for the lentigines.^3^ In addition, Wang et al. reported that IL-17 and TNF can affect both the growth and pigment production of melanocytes, which may contribute to the pigmentation changes associated with psoriasis.^4^ In turn, it has been suggested that eruptive lentiginosis is an exaggerated recovery in pigment production, associated with greater disease severity or greater inhibition of cytokines with treatment.^1^

To date, no effective therapy has been reported. Lentigines appear within the first months of treatment and may persist with no or little improvement.^1^ Although it does not require an interruption of the treatment, close follow-up is recommended.

In conclusion, the author presents a new case of eruptive lentiginosis confined to areas of regressing psoriatic plaques after adalimumab. Given the development of novel biological treatments and new therapeutic targets, new cases of eruptive lentiginosis are likely to appear. Clinicians need to be aware of the potential side effects of biological therapies due to their increasing use.

## Financial support

None declared.

## Author's contributions

Fernando Garcia-Souto: Approval of the final version of the manuscript; design and planning of the study; drafting and editing of the manuscript; critical review of the literature; critical review of the manuscript.

## Conflicts of interest

None declared.
